# Identification of pandemic clade-specific genetic marker with genomic insight into Vibrio parahaemolyticus

**DOI:** 10.1099/acmi.0.001067.v4

**Published:** 2026-02-26

**Authors:** Masatomo Morita, Kazuhisa Okada, Sarunporn Tandhavanant, Hirotaka Hiyoshi, Eiji Arakawa, Hidemasa Izumiya, Amonrattana Roobthaisong, Warawan Wongboot, Moses Lorenzo Akyeh, Tetsuya Iida, Yukihiro Akeda, Toshio Kodama

**Affiliations:** 1 Department of Bacteriology I, National Institute of Infectious Diseases, Tokyo, Japan; 2 Thailand-Japan Research Collaboration Center on Emerging and Re-emerging Infections, Osaka University, Nonthaburi, Thailand; 3 Research Institute for Microbial Diseases, Osaka University, Osaka, Japan; 4 Department of Bacteriology, Institute of Tropical Medicine, Nagasaki University, Nagasaki, Japan; 5 Department of Microbiology and Immunology, Faculty of Tropical Medicine, Mahidol University, Bangkok, Thailand; 6 Department of Medical Sciences, National Institute of Health, Nonthaburi, Thailand; 7 Programme for Nurturing Global Leaders in Tropical and Emerging Communicable Diseases, Graduate School of Biomedical Sciences, Nagasaki University, Nagasaki, Japan; 8 Noguchi Memorial Institute for Medical Research, University of Ghana, Accra, Ghana

**Keywords:** genomic island, genetic marker, pandemic clade, *Vibrio parahaemolyticus*, whole-genome sequencing

## Abstract

*Vibrio parahaemolyticus* is a foodborne pathogen commonly present in seafood. Of the various *V. parahaemolyticus* serotypes reported, O3:K6, O1:K25, O1:KUT and O4:K68 represent the major serotypes among pandemic clones that emerged from 1995 onward. However, new molecular markers of pandemic clones remain unidentified, and limited genomic sequence data are available for non-pandemic strains. Therefore, we aimed to identify novel genetic markers specific to pandemic *V. parahaemolyticus* strains by comparing non-pandemic and pandemic strains using whole-genome sequencing. Phylogenetic analysis of 163 *V*. *parahaemolyticus* strains revealed high genomic diversity within the species. The analysis also revealed a pandemic clade consisting of serotypes O3:K6, O1:K25, O1:KUT and O4:K68 strains isolated after 1995. We identified the genomic island GI-110 (VPaI-5) as a potential marker exclusive to the pandemic clade. Multiplex PCR detection of VPaI-5 demonstrated high specificity for pandemic strains, outperforming the detection of existing markers. The capacity of multiplex PCR for VPaI5 in distinguishing between pandemic and non-pandemic strains was confirmed using clinical isolates from Thailand. Our findings provide valuable insights into the genetic diversity of *V. parahaemolyticus* and establish a reliable method for monitoring pandemic strains.

**Impact Statement**

This study addresses the need for the accurate detection of pandemic *Vibrio parahaemolyticus* strains responsible for global foodborne outbreaks. The identification of novel genetic markers specific to pandemic strains has guided the development of detection assays for these strains. VPaI5-PCR, which specifically detects the pandemic clade of *V. parahaemolyticus*, offers a reliable tool for routine food safety testing, environmental monitoring and public health surveillance.

## Data Summary

Short-read sequence data were submitted to the DDBJ Sequenced Read Archive, and the accession numbers are listed in Tables S1 and S3.

## Introduction

*Vibrio parahaemolyticus* is a normal inhabitant of marine, estuarine and coastal environments; it causes foodborne infections, particularly those associated with seafood consumption. *V. parahaemolyticus* infections were first reported during an outbreak in Japan in 1950, followed by sporadic cases involving various serotypes in geographically diverse locations [1]. However, after a novel O3:K6 strain emerged in 1995, strains of the O3:K6, O1:K25, O1:KUT and O4:K68 serotypes have emerged as major causative agents worldwide [2,4]. Because they share common molecular characteristics, these isolates were considered as serovariants of the original O3:K6 strain, and all of them, including the O3:K6 strain, are referred to as the *V. parahaemolyticus* pandemic clone [3].

The need for rapid testing of food, faecal and environmental samples has led to the identification of biomarkers for screening pandemic clones. Several studies have reported genetic and protein markers unique to the pandemic clone, which has resulted in the development of PCR-based detection methods [5,8]. Although numerous *V. parahaemolyticus* genomes have been sequenced in the post-genomics era, no studies have been undertaken to reassess the biomarkers and identify new pandemic markers [9]. Further, limited sequenced genomic data are available for non-pandemic strains, and comparative genomics between non-pandemic and pandemic strains has not been examined. The primary objective of this study is to improve the rapid and accurate identification of pandemic *V. parahaemolyticus* strains using comprehensive genomic data. Therefore, we performed whole-genome sequencing (WGS) of both non-pandemic and pandemic strains and identified several genomic islands (GIs), one of which is unique to pandemic strains, which can be used as a novel genetic marker for the pandemic strains. Furthermore, we developed a novel multiplex PCR assay targeting VPaI-5, enabling highly specific detection of pandemic strains.

## Methods

### Bacterial strains and WGS

The *V. parahaemolyticus* strains used in this study were obtained from the Pathogenic Microbes Repository Unit, Research Institute for Microbial Diseases (RIMD), Osaka University. A total of 162 *V*. *parahaemolyticus* strains from the RIMD collection were selected for WGS while avoiding duplication of serotypes and isolation years (Table S1, available in the online Supplementary Material). Genomic DNA was extracted using the DNeasy Blood and Tissue Kit (Qiagen, Hilden, Germany), and concentrations were determined using Qubit dsDNA HS Assay Kit (Thermo Fisher Scientific, Waltham, MA, USA). For WGS, a genomic library was prepared using the Nextera XT DNA Library Preparation Kit (Illumina, San Diego, CA, USA) and sequenced on NovaSeq 6000 (Illumina) to generate 250 bp paired-end reads.

To confirm the performance of a novel PCR-based pandemic marker, described below, WGS of 71 clinical isolates from Thailand was performed on MiSeq (Illumina) with 300 bp paired-end reads, using a genomic library prepared by Illumina DNA Prep (Illumina), and 118 draft genome sequences of clinical isolates were retrieved from the DDBJ/ENA/GenBank database (Tables S3 and S4) [10,13].

### Genome assembly and annotation

Genome assembly was performed using SPAdes v.3.13.0 with the ‘--careful’ and ‘--cov-cutoff auto’ options [14] after processing of the raw reads using fastp v.0.20.1 [15]; contigs less than 500 bp in length were removed from the draft genome. The reference genome of RIMD2210633 (DDBJ/ENA/GenBank accession numbers: BA000031.2 and BA000032.2) and 118 draft genome sequences of clinical isolates from the public database were included in further analysis (Table S4) [10,13,16]. Draft and reference genomes were annotated using Bakta v.1.11 [17]. Default parameters were used in all software programmes unless otherwise specified.

### Pan-genome and phylogenetic analysis

Pan-genomes and core gene alignments were constructed using Panaroo v.1.5.0 with the ‘--clean-mode strict’, ‘--merge_paralogs’, ‘-a core’ and ‘--aligner mafft’ options [18]. Single nucleotide variants (SNVs) were extracted from the core gene alignment using SNP-sites v.2.5.1 and used to reconstruct a phylogenetic tree using IQ-TREE v.2.0.3 with 1,000 ultrafast bootstrap replicates [19,20]. The midpoint-rooted phylogenetic tree was visualized using interactive Tree of Life (https://itol.embl.de/) [21]. Strains from the RIMD collection and other isolates were analysed separately. The SNV alignment was also applied for the Bayesian analysis of population structure (BAPS) using fastbaps v.1.0.8, which was implemented in R package. The analysis was performed with the function ‘multi_res_baps’, using the ‘optimize.baps’ prior and two levels of clustering [22].

### Identification and distribution of GIs

We defined GIs as regions more than 10 kb in length between the two loci of core genes or tRNAs in the complete genome sequence of RIMD2210633, which contained more than two CDSs. The core genes (present in ≥95% of strains) were extracted from the pan-genome profile of strains from the RIMD collection, and tRNAs of strain RIMD2210633 were annotated according to Bakta as mentioned above. The presence or absence of CDSs on the GIs in the genome of each isolate was confirmed from the pan-genome profiles.

### *In silico* PCR of pandemic markers

We performed *in silico* PCR to detect pandemic markers using primer pair sequences for GS-PCR, *orf8*-PCR and PGS-PCR (Table S2) [5,7], with thresholds of 98% identity and a minimum length of 98% between each primer and genome sequence. A positive result was obtained when the fragment size was the same as expected. The *in silico* VPaI5-PCR process described below was performed in the same manner.

### Development of a novel pandemic marker

Multiplex PCR was performed to determine the presence or absence of VPaI-5, which was named VPaI5-PCR (Fig. S1). The primer sequences used for VPaI5-PCR are listed in Table S2. To assess whether these primers could successfully detect the *V. parahaemolyticus* pandemic clone, we used the genomic DNA of clinical *V. parahaemolyticus* isolates from Thailand as a template for VPaI5-PCR. The performance was evaluated in terms of sensitivity and specificity. Sensitivity was defined as the proportion of true-positive samples correctly identified by the assay, calculated as the number of true positives divided by the sum of true positives and false negatives. Specificity was defined as the proportion of true-negative samples correctly identified by the assay, calculated as the number of true negatives divided by the sum of true negatives and false positives.

## Results

### Population structure of *V. parahaemolyticus*

**Fig. 1.**
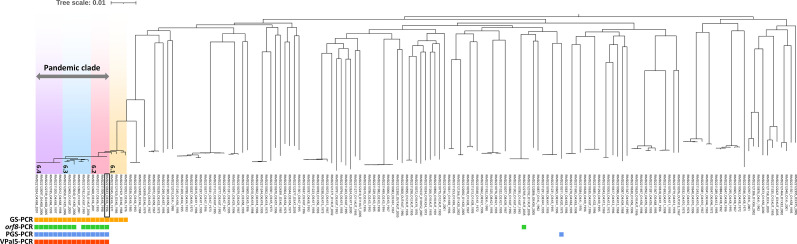
Population structure of *V. parahaemolyticus* based on the core gene alignment and specificity of pandemic markers. Serotype and year of isolation are indicated after the strain name. Subclusters 6.1, 6.2, 6.3 and 6.4, identified through BAPS using fastbaps v.1.0.8, are highlighted in different colours. Positive results for the pandemic markers are indicated using squares at the bottom. The pandemic reference strain RIMD2210633 is boxed in a square.

We evaluated the phylogenetic relationships between *V. parahaemolyticus* strains and inferred their population structures using BAPS (Fig. 1). BAPS classification revealed that the 163 *V*. *parahaemolyticus* strains were assigned into 18 clusters at level 1. Twenty strains, including the reference genome of the pandemic clone (RIMD2210633), were assigned to BAPS cluster 6 (Table S1). BAPS cluster 6 comprised both non-pandemic and pandemic strains, which were further subclassified into four subclusters at level 2 and designated as BAPS subclusters 6.1, 6.2, 6.3 and 6.4 (Fig. 1). BAPS subcluster 6.1 comprised only the non-pandemic O3:K6 strains isolated between 1971 and 1988. In contrast, BAPS subclusters 6.2, 6.3 and 6.4 consisted of pandemic strains that were concordant with the serotypes. Subcluster 6.2 comprised O3:K6 serotype strains, subcluster 6.3 comprised serogroup O1 strains with three different K serotypes and subcluster 6.4 comprised O4:K68 serotype strains. These three subclusters (6.2, 6.3 and 6.4) could be defined as the *V. parahaemolyticus* pandemic clade. To reassess the specificity and sensitivity of three previously reported pandemic markers (GS-PCR, *orf8*-PCR and PGS-PCR), we conducted *in silico* PCR analyses. The results revealed inconsistencies: several strains outside the pandemic subclusters were positive, whereas some strains within the subclusters were negative (Fig. 1) [5,7]. GS-PCR yielded positive results for all non-pandemic strains of BAP subcluster 6.1, which was nearest to the pandemic clade, in addition to the strains of the pandemic clade. The *orf8*-PCR results were positive for all but one strain in the pandemic clade and also positive for one O1:KUT strain in BAPS subcluster 13.1 [23]. When PGS-PCR was used, the pandemic strains within the BAPS cluster 6 tested positive; however, a genetically distant serotype O8:K20 strain BAPS subcluster 18.7, isolated in 1975, was also positive. We then attempted to identify the genetic elements that existed only in the strains of the pandemic clade that were used as novel pandemic markers.

### GIs specific to the pandemic clade

**Fig. 2.**
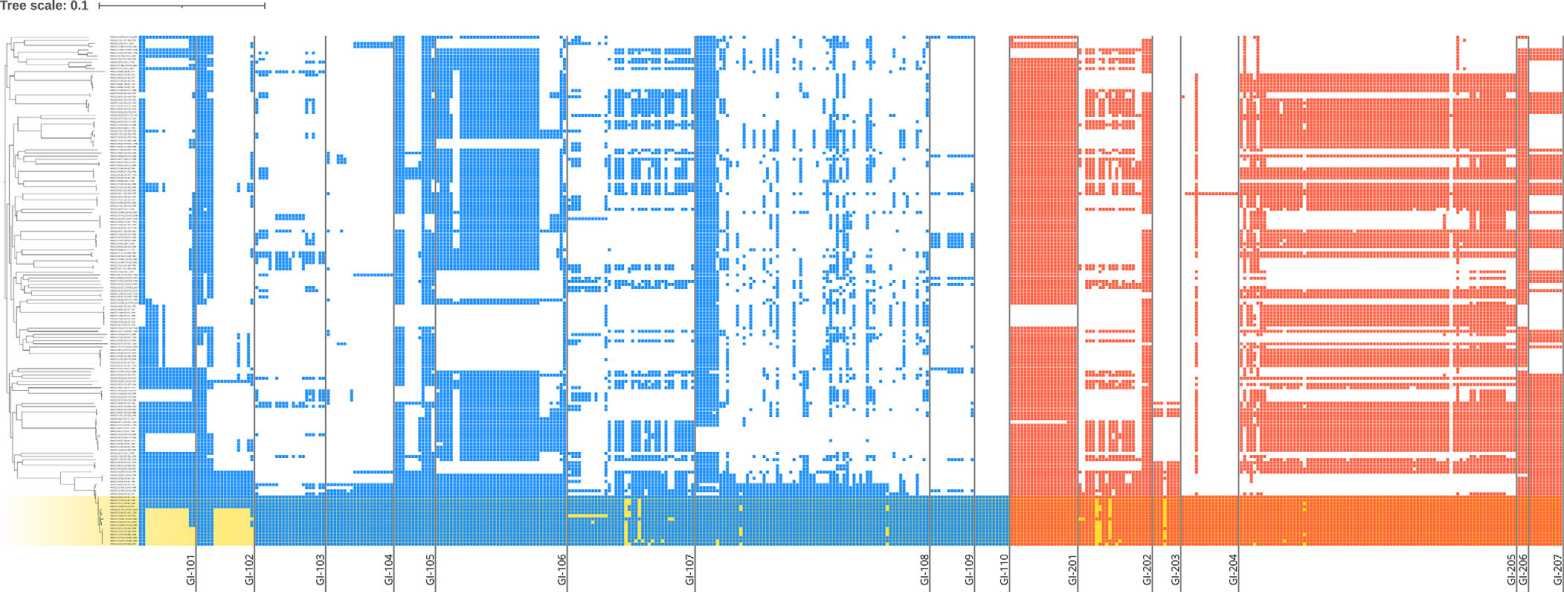
Distribution of CDSs on GI. Strains of pandemic clade (BAPS subclusters 6.2, 6.3 and 6.4) are highlighted in yellow. Blue boxes and red boxes represent CDSs on GI in chromosome 1 and chromosome 2, respectively.

GIs play an important role in the genomic diversification of *Vibrio* spp., and it is likely that strains of the pandemic clade acquired specific GIs during evolution [24]. Therefore, we explored the GIs unique to pandemic clade strains to identify novel pandemic markers. We identified 17 GIs in the RIMD2210633 genome, which included the O- and K-antigen biosynthetic gene clusters, type 6 secretion system gene cluster and *V. parahaemolyticus* islands (VPaIs) (Table 1) [25]. To determine which GIs were specific to the pandemic clade, the distribution of CDSs on GIs was examined using pan-genome profiles. GI-110 was present only in the pandemic clade (Fig. 2). GI-110, also known as VPaI-5, consists of 11 CDSs encoding 1 integrase and 10 hypothetical proteins [25].

**Table 1.** Statistics of the 17 GIs identified in the *V. parahaemolyticus* strain RIMD2210633 complete genome

GI	Chromosome	Start	End	Size	No. of CDS	Remark
GI-101	1	206021	222494	16,474	17	O-antigen genes
GI-102	1	230328	249504	19,177	17	K-antigen genes
GI-103	1	381054	403433	22,380	21	VPaI-1 (381054–403433)*
GI-104	1	1121252	1143500	22,249	20	VPaI-3 (1121252–1152668)*
GI-105	1	1441204	1454554	13,351	12	
GI-106	1	1468883	1527984	59,102	39	Type VI secretion system genes
GI-107	1	1658752	1680208	21,457	35	
GI-108	1	1894516	1947999	53,484	70	Superintegron
GI-109	1	2240007	2256166	16,160	13	VPaI-4 (2240007–2256166)*
GI-110	1	3084855	3100003	15,158	10	VPaI-5 (3084846–3099979)*
GI-201	2	741193	762779	21,587	20	
GI-202	2	933840	947590	13,751	25	
GI-203	2	984410	1000321	15,912	8	
GI-204	2	1326614	1352643	26,030	17	VPaI-6 (1325821–1352643)*
GI-205	2	1388102	1467625	79,524	84	VPaI-7 (1390967–1501509)*
GI-206	2	1518749	1534543	15,795	3	
GI-207	2	1818647	1829198	10,552	10	

*VPaIs were reported by Hurley CC, *et al*. BMC Genomics (2006) 7:104.

### Performance of the novel genetic marker

Although its functions have not yet been elucidated, a PCR-based method was developed for detecting GI-110 as a potential tool for identifying pandemic clones. Two sets of primer pairs were designed: one consisted of a forward primer within the chromosomal core gene adjacent upstream of the integrase gene on VPaI-5 and a reverse primer within the integrase gene on VPaI-5, and the other pair consisted of a forward primer within the most downstream gene of VPaI-5 and a reverse primer within the chromosomal core gene adjacent downstream of VPaI-5 (upstream and downstream, respectively; Table S2). These primer sets in the multiplex PCR would amplify two or one fragment in VPaI-5-positive or -negative strains, respectively (Fig. S1). This method exhibited 100% specificity and 100% sensitivity across 163 *V*. *parahaemolyticus* strains from the RIMD collection.

**Fig. 3.**
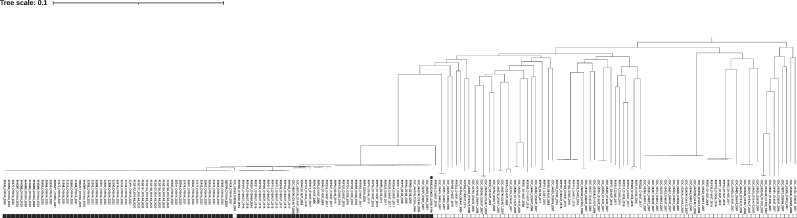
Phylogenetic tree of clinical isolates from Thailand with the results of VPaI5-PCR. Serotype and year of isolation are indicated after the strain name. The pandemic reference strain of RIMD2210633 (marked with a circle) was included in the phylogenetic analysis. Positive and negative results of VPaI5-PCR were shown in filled and outlined boxes, respectively. The results of Thai isolates and RIMD2210633 were derived from the multiplex PCR assay, while those of isolates from the public database were derived from *in silico* PCR.

We used 71 clinical *V. parahaemolyticus* isolates from Thailand to confirm the performance of VPaI5-PCR (Tables S3), and 118 genomes of clinical isolates from public databases were subjected to *in silico* analysis of VPaI5-PCR (Table S4). In the phylogenetic tree, including the pandemic reference strain RIMD2210633, 103 strains were clustered in the same clade as the pandemic reference strain, and all except for one strain were tested positive with two amplicons at 827 and 649 bp (Fig. 3). In contrast, the other 87 strains showed diverse locations but were outside the pandemic clade on the phylogenetic tree and tested negative with one amplicon of 468 bp in size. Unlike PCR for other pandemic strain markers, VPaI5-PCR yielded positive results only for strains of the pandemic clade (BAPS subclusters 6.2, 6.3 and 6.4), demonstrating 99.0% sensitivity and 100% specificity (Fig. 1). VPaI5-PCR was the most specific method for the pandemic clade.

## Discussion

The emergence of the pandemic *V. parahaemolyticus* serotype O3:K6 and its serovariants is a global public health concern owing to its association with seafood-borne illnesses. In this study, we reassessed previously reported genetic markers to identify novel markers unique to the pandemic clade by comparing non-pandemic and pandemic strains via WGS. Further, we developed a PCR-based detection method, leading to surveillance of pandemic *V. parahaemolyticus* and prevention of associated foodborne outbreaks.

Currently, GS-PCR targets the pandemic-type *toxRS* and is the most commonly used method since its initial report in 2000 [5]; isolates positive for both the thermostable direct haemolysin gene (*tdh*) and GS-PCR are considered as pandemic strains. The method has not undergone systematic reevaluation, even in the post-genomic era, and no alternative methods have been proposed [26]. However, reassessment based on the phylogenetic analyses of this study has shown that four strains of the BAPS subcluster 6.1 also possess the pandemic-type *toxRS*. Notably, the four strains are considered non-pandemic, since they were isolated before the emergence of pandemic strains (in 1971, 1976, 1986 and 1988). This suggests that a GS-PCR result can lead to false positives and is not sufficient for identifying a pandemic clone [27]. Furthermore, strains within the pandemic clade that tested negative for *orf8*-PCR were observed. This is consistent with previous reports and suggests that *orf8*-PCR is also an insufficient pandemic marker [23]. Since *orf8* is encoded by a lysogenized filamentous phage, it may be lost during phage induction. Consequently, the phage may infect and lysogenize non-pandemic strains, suggesting that *orf8* has low stability as a pandemic marker. Prior to this study, the PGS-PCR amplicon sequence was considered specific to pandemic strains [28]. However, our genomic overview of *V. parahaemolyticus* revealed the presence of a PGS-PCR-positive non-pandemic strain. Additionally, PGS-PCR targets a genomic region on chromosome 2. In *Vibrio* spp., chromosome 2 contributes to genome diversification both as a donor and recipient of genetic elements [25]. Therefore, the region targeted by PGS-PCR is considered more susceptible to mutations than the genomic region on chromosome 1, and it potentially spreads to non-pandemic isolates via horizontal gene transfer. Therefore, the use of VPaI-5, which is located on a relatively stable chromosome 1, as a pandemic marker is considered a good approach owing to its high specificity.

VPaI-5, a 16 kb insertion sequence originally identified within the gene encoding the histone-like DNA-binding protein HU-*α*, induces a frameshift mutation and has been characterized as a specific region in the pandemic clone of *V. parahaemolyticus* [28]. In our study, one strain (CDC_K5528) within the pandemic clade was found to be negative for VPaI-5, despite its clustering phylogenetically with other VPaI-5-positive pandemic strains. Similarly, an O3:K6 strain isolated in Mexico in 2009, which lacked VPaI-5 but retained other core genomic elements associated with the pandemic clone [29]. These findings suggest that VPaI-5, although generally conserved, may undergo integrase-mediated excision or be affected by genomic rearrangement, leading to its absence in certain strains. However, both the VPaI-5-negative isolates above were TDH-negative and thus are classified as non-pandemic strains under the current criteria. In the present study, we developed a novel PCR test, VPaI5-PCR, which can specifically and effectively distinguish pandemic clade strains from non-pandemic strains, as it generates two amplicons in a positive test and one in a negative test. This prevents false-positive results and enables easy detection of sample contamination. Our findings highlight the genetic diversity within *V. parahaemolyticus* and provide a basis for implementing VPaI5-PCR in the routine testing of food, faecal and environmental samples. However, it remains unclear whether VPaI-5 acts as a factor in the pandemic potential of *V. parahaemolyticus*. Further studies should characterize the functional roles of VPaI-5 and continue to monitor pandemic *V. parahaemolyticus* strains as a potential public health threat.

## Supplementary material

10.1099/acmi.0.001067.v4 Uncited Supplementary Material 1.

10.1099/acmi.0.001067.v4 Uncited Supplementary Material 2.

## Abbreviations

BAPS: Bayesian analysis of population structure
GI: genomic island
RIMD: Research Institute for Microbial Diseases
SNV: single nucleotide variant
VPaI: *Vibrio parahaemolyticus* island
WGS: whole-genome sequencing
