# Supplementary material for: Identification of pandemic clade-specific genetic marker with genomic insight into Vibrio parahaemolyticus
Source: Access Microbiol. 2026 Feb 26;8(2):001067.v4. doi: 10.1099/acmi.0.001067.v4 (PMC12945327; doi:10.1099/acmi.0.001067.v4)
Supplement: Uncited Supplementary Material 1. [file acmi-8-01067-s001.pdf]

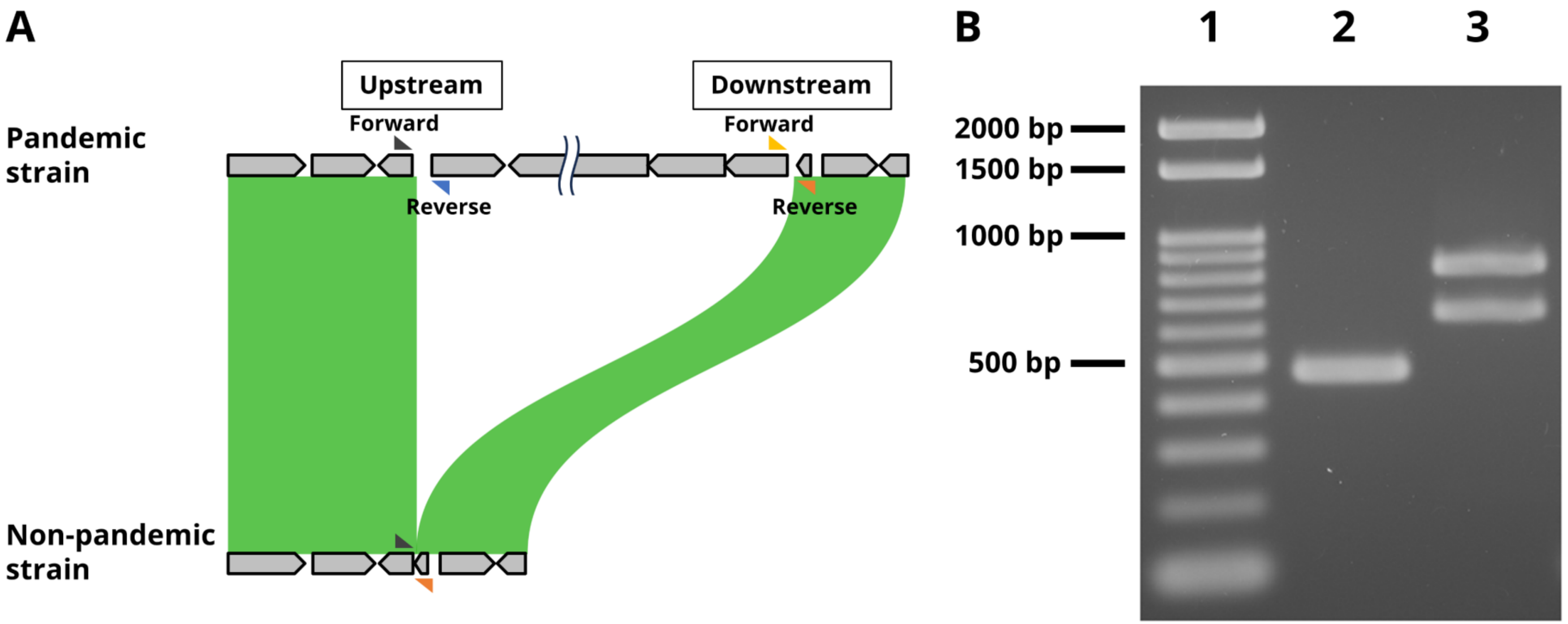

Fig. S1. Schematic summary of VPaI5-PCR primer design (A) and agarose gel electrophoresis of the amplicons (B). (A) Triangles show position of each primer. In pandemic strains, both the Upstream and Downstream primer pairs anneal to their respective target sequences, yielding two amplicons. In non-pandemic strains, only the Upstream forward primer and the Downstream reverse primer anneal, resulting in the generation of a single amplificon. (B) Lane 1: 100bp DNA ladder marker, lane 2: one amplicon from non-pandemic strain RIMD2210345, lane 3: two amplicons from pandemic strain RIMD2210633.
